# Lithium Accumulates in Neurogenic Brain Regions as Revealed by High Resolution Ion Imaging

**DOI:** 10.1038/srep40726

**Published:** 2017-01-18

**Authors:** Giulia Zanni, Wojciech Michno, Elena Di Martino, Anna Tjärnlund-Wolf, Jean Pettersson, Charlotte Elizabeth Mason, Gustaf Hellspong, Klas Blomgren, Jörg Hanrieder

**Affiliations:** 1 Karolinska Institute, Department of Women’s and Children’s Health, Karolinska University Hospital, Stockholm, Sweden; 2 Center for Brain Repair and Rehabilitation, Institute of Neuroscience and Physiology, Sahlgrenska Academy at the University of Gothenburg, Gothenburg, Sweden; 3 Department of Psychiatry and Neurochemistry, Institute of Neuroscience and Physiology, Sahlgrenska Academy at the University of Gothenburg, Mölndal, Sweden; 4 Clinical Neurochemistry Laboratory, Sahlgrenska University Hospital, Mölndal, Sweden; 5 Department of Chemistry-Biomedical Centre, Uppsala University, Uppsala, Sweden; 6 Department of Pediatric Oncology, Karolinska University Hospital, Stockholm, Sweden; 7 Department of Chemistry and Chemical Engineering, Chalmers University of Technology, Gothenburg, Sweden; 8 Department of Molecular Neuroscience, UCL Institute of Neurology, University College London, London, United Kingdom

## Abstract

Lithium (Li) is a potent mood stabilizer and displays neuroprotective and neurogenic properties. Despite extensive investigations, the mechanisms of action have not been fully elucidated, especially in the juvenile, developing brain. Here we characterized lithium distribution in the juvenile mouse brain during 28 days of continuous treatment that result in clinically relevant serum concentrations. By using Time-of-Flight Secondary Ion Mass Spectrometry- (ToF-SIMS) based imaging we were able to delineate temporospatial lithium profile throughout the brain and concurrent distribution of endogenous lipids with high chemical specificity and spatial resolution. We found that Li accumulated in neurogenic regions and investigated the effects on hippocampal neurogenesis. Lithium increased proliferation, as judged by Ki67-immunoreactivity, but did not alter the number of doublecortin-positive neuroblasts at the end of the treatment period. Moreover, ToF-SIMS revealed a steady depletion of sphingomyelin in white matter regions during 28d Li-treatment, particularly in the olfactory bulb. In contrast, cortical levels of cholesterol and choline increased over time in Li-treated mice. This is the first study describing ToF-SIMS imaging for probing the brain-wide accumulation of supplemented Li *in situ*. The findings demonstrate that this technique is a powerful approach for investigating the distribution and effects of neuroprotective agents in the brain.

Lithium (Li) is a potent mood stabilizer used in the treatment of bipolar disorder. The therapeutic window is not wide, ranging from 0.6 to 1.2 mmol/L Li in serum, making regular monitoring of the plasma concentrations in patients important^1^. Interestingly, the lithium ion has a similar atomic and ionic radius as magnesium and accordingly it is believed to act as a second messenger, inhibiting a broad range of enzymatic reactions^2^. Hence, numerous signaling pathways are targeted, including IP3^2^,^3^, which in turn modulates a series of other downstream pathways^4^,^5^. The most widely investigated mechanism is GSK3β and the downstream activation of this well-known pathway^6^,^7^,^8^. Despite the wide use of lithium in medical treatment and extensive research carried out during the past decades, the exact molecular mechanisms of action are not yet elucidated. Lithium has proven to be potent in enhancing hippocampal neurogenesis in adult as well as in young^9^ mice after cranial irradiation, at least partially due to inhibition of apoptosis^10^. Generation of new neurons occurs in two discrete regions of the postnatal brain, the subventricular zone (SVZ) and the subgranular zone (SGZ) of the dentate gyrus (DG) in the hippocampus^11^,^12^. Importantly, hippocampal neurogenesis seems to be essential for cognition, particularly in encoding of new memories^13^,^14^. Possible age-dependent differences in the pharmacokinetics and pharmacodynamics of lithium prompt further investigation of its distribution and effects in the developing brain, especially when considering lithium as a potential treatment for cognitive and degenerative disorders in children.

Molecular imaging is of central importance in order to obtain spatial and temporal information on molecular concentration changes *in vivo* or *in situ*. Particularly, mass spectrometry-based molecular imaging (MSI) has been demonstrated to be a valuable approach in biomedical research. It represents a powerful technology for comprehensive spatial profiling of various chemical species, including inorganics, drugs, metabolites, lipids, neuropeptides and proteins in complex biological matrices^15^,^16^,^17^. In contrast to common molecular and histological imaging techniques, MSI does not require any *a priori* knowledge of the potential target species. An integral advantage of time-of-flight secondary ion mass spectrometry- (ToF-SIMS) based imaging is the potential of mapping inorganic and organic chemical species in biological tissues and cells. The technique is fairly reproducible (CV 10%) as evaluated recently in an inter-laboratory study on reference materials^18^. ToF-SIMS features high spatial resolution, often at the submicron scale (<500 nm), making it a powerful technology for chemical imaging at the single cell level^19^. This is of particular relevance when studying complex and heterogeneous samples, such as brain tissue, which arguably constitutes the most complex and least understood system in the body^20^,^21^,^22^. Previous studies on adult rodent brain showed that once lithium reaches a steady state, it displays a regional distribution in the brain, and by using a neutron radiation technique the highest lithium concentrations were observed in the thalamus, neo-cortex, the gray matter of the cerebellum and the hippocampus^23^,^24^,^25^.

In the present study, a novel approach based on ToF-SIMS imaging was employed for the first time to probe the temporospatial accumulation of lithium and associated changes of low molecular weight species (<1,000 Da) *in situ*. The technique was used to monitor brain wide lithium and lipid changes in 2-month-old mice during 28 days of lithium treatment. We further compared the effects of stable lithium levels, within the therapeutic range, on neural stem/progenitor cell (NSPC) proliferation and differentiation in the juvenile versus the adult mouse hippocampus.

## Materials and Methods

### Chemicals and Reagents

All chemicals were of pro-analysis grade and purchased from Sigma Aldrich. TissueTek optimal cutting tool (OCT) was purchased from Sakura Finetek (AJ Alphen aan den Rijn, The Netherlands). Water was purified with a Milli-Q (Millipore, Bedford, MA, USA) purification system.

### Animals and ethical permission

In all experiments C57BL/6 female mice from Charles Rivers Laboratories were used (Sulzfeld, Germany). The animals were kept under standard conditions of daylight (12 hour light cycle) and provided food and water *ad libitum*. All experimental conditions were approved by the Animal Research Ethics Committee in Gothenburg), in accordance with the national animal welfare legislation (application no. 20-2013 and N248-13). Animals were delivered with their respective dams and weaned on postnatal day (PND) 21 or PND 49 for adult experiments. In total 135 animals were used in this study, divided in different experiment as follows: 24 mice (equally divided between lithium treated (12: 4 × 3/group) and controls (12: 4 × 3/group)) for ToF-SIMS, 4 mice (only lithium treated) for ICP-AES, 3 (only lithium treated) adult and 94 (only lithium treated) young mice for serum lithium determination, 10 adult (equally divided between lithium treated and controls) and 12 (equally divided between lithium treated and controls) young mice for immunohistochemistry (IHC).

### Lithium administration

On PND 21 or PND 49, female littermates (5–6 animals in each cage) were randomly assigned to lithium or control treatment. Prior to the administration of the food pellets, animals received either a 4 mmol/kg loading dose of LiCl (Sigma Aldrich, St. Louis, USA) or 0.9% NaCl (vehicle) intraperitoneally. Immediately after the injection, lithium (2.4 g/kg Li_2_CO_3_, TD.05357, Harlan laboratories, Netherlands) or control chow (T.2918.CS, Harlan laboratories, Netherlands) was provided. To minimize the side effects of lithium on kidney function, a saline bottle was provided in all lithium as well as control cages, as previously reported^26^. The mean food intake was calculated by measuring the amount of food consumed daily in each cage (n = 2–3) and dividing this by the number of animals (n = 5–6) per cage.

### Determination of lithium in blood and brain

Blood samples were collected at different time points via transcutaneous cardiac puncture and the serum separated for determination of lithium levels. The Clinical Chemistry Department at the Sahlgrenska University hospital performed the lithium serum analysis using a Roche/Hitachi cobas 8000, c502 analyzer.

For brain tissue lithium determination, micro-dissection of defined brain regions was performed after 28 days of lithium treatment and the tissue pieces were snap frozen in dry ice and kept in −80. Neurogenic regions where delineated as previously described^27^,^28^,^29^. Each region was carefully weighed and homogenized with an ultrasonicator (Vibra-cell™, VC750, Sonics & Materials, Inc., Danbury, CT, USA) in 10 volumes of 0.5 N trichloroacetic acid (TCA) followed by centrifugation. For each sample 50 μl was further diluted in 4.95 ml of 0.02 M HNO_3_ and the lithium concentrations in parts per million (ppm) were measured using inductively coupled plasma atomic emission spectroscopy (ICP-AES) (Spectro Ciros^CCD^ EOP, Spectro Analytical Instruments, Kleve, Germany). The lithium concentrations were quantitatively determined by using the emission at 670.780 nm against acid-matched calibration solutions and the concentration (μg/g tissue) was calculated from the dilution factor (100) and the respective weight in grams for each tissue sample (Supplementary Fig. 1).

### Tissue Sample Preparation for ToF-SIMS Analysis

Animals were sacrificed by decapitation following isoflurane anesthesia at 2, 7, 14 or 28 days after the onset of lithium administration. A number of three animals was analyzed per group. Brains were rapidly excised; snap frozen in dry ice and isopentane followed by storage at −80 °C. Brains were cryosectioned sagittally and collected in consecutive series using a cryostat microtome (Leica Biosystems, Nussloch, Germany) at −18 °C. The tissue sections (12 μm) were thaw mounted on Superfrost glass slides (Thermo Scientific), dried under vacuum for 10 min and stored at −20 °C until use.

ToF-SIMS analysis was performed on an ION-TOF V instrument (ION-TOF GmbH, Münster, Germany) using a Bi^3+^ cluster ion gun as primary ion source. All spectra were acquired and processed with the Surface Lab software (v. 6.3 ION-TOF). Data from one single sagittal per animal section were acquired in high current bunched (HCB) mode with a pulsed primary ion current of 0.28 pA at 25 keV and a maximum ion dose density was 2 × 10^9 ^ions/cm^2^. Scans were acquired in positive mode using the stage “scan macro raster function” with 10 shots per pixel on 0.4 mm × 0.4 mm areas (patches) with 200 measurements per mm resulting in a pixel resolution of 5 μm. This acquisition mode comprises stepwise acquisition patches over the whole tissue area in electrostatic raster mode and stepwise movement of the sample stage. The mass resolution in HCB mode was about M/∆M = 5 × 10^3^. All spectra were calibrated internally to signals of [C]^+^, [CH]^+^, [CH_2_]^+^, [CH_3_]^+^, [C_5_H_15_PNO_4_]^+^ and [C_27_H_45_]^+^. A mass interval list was created by peak search of all individual samples according to the following search parameters: S/N > 3, width 0.8 Da.

### Immunohistochemistry

Another set of animals was used for stereological quantification of doublecortin (DCX), BrdU (5-Bromo-2′-Deoxyuridine) and Ki67. Adult animals were injected intraperitoneall with BrdU (B5002, Sigma-Aldrich, St. Louis, MO, USA) at 50 mg/kg for five days from PND59. After 14 or 28 days of lithium treatment, animals were deeply anesthetized with pentobarbital and saline solution (0.9% NaCl) was delivered transcardially through the left ventricle, followed by 4% paraformaldehyde (PFA). Brains were removed, keeping the olfactory bulb intact, and after 24 hours transferred into 30% sucrose in 0.1 M phosphate buffer. Brains were cryosectioned sagittally in 1:12 consecutive series of 25 μm thick sections on a sliding microtome (Leica SM 2000R) and kept in a cryoprotectant solution of 25% ethylene glycol, 25% glycerol and 0.1 M phosphate buffer (pH 7.4) at +4 °C. For chromogenic immunodetection (3,3-diaminobenzidine (DAB) staining) free-floating sections were washed in Tris-buffered saline (TBS) for 30 minutes (Trizma HCl, Trizma base, NaCl, pH 7.5) and treated with 0.6% H_2_O_2_ for 30 minutes. Antigen retrieval was performed by heating the slides in sodium citrate buffer (10 mM, pH 6.0) at 80 °C for 30 minutes. For BrdU detection, antigen retrieval consisted of incubation in 2 N HCl for 30 minutes at room temperature followed by 0.1 M borate buffer pH 8.5 at room temperature for 10 minutes. Sections were then incubated in blocking solution (0.1% Triton X-100, 3% donkey serum in TBS) for 1 hour at room temperature on a shaking plate followed by primary antibody for 72 hours and incubation in avidin-biotin peroxidase solution (Vectastain ABC Elite kit, Vector Laboratories, Burlingame, CA), DAB and 0.04% NiCl_2_. The secondary antibodies were diluted 1:1,000 and kept for 1 hour at room temperature on a shaking plate. The following primary antibodies were used: mouse monoclonal anti-Ki67 (1:100, Leica Biosystems, Kidlington, UK), goat anti- DCX (1:100, Santa Cruz Biotechnology Inc., CA, USA) and rat anti-BrdU (1:500, AbD Serotec, Kidlington, UK). The following secondary antibodies were used: biotinylated donkey anti-goat IgG (H + L), biotinylated donkey anti-rat IgG (H + L) and biotinylated donkey anti-mouse IgG (H + L) (Jackson ImmunoResearch Europe, Suffolk, UK). Sections were dehydrated with NeoClear^®^ and mounted in NeoMount^®^ (Millipore Merck KGaA, Darmstadt, Germany).

### Counting procedures

A series of every 12^th^ section was examined to count the total number of DCX^+^, Ki67^+^ and BrdU^+^ cells in the granule cell layer (GCL) across all hippocampal sections of that series. The areas of the GCL were measured to calculate the volume and enable cell density calculations. The total number of cells was assessed and contour areas created using unbiased counting software (Stereo Investigator, MicroBrightField Inc.; Colchester, VT, USA).

### Data Analysis

For image analysis, the whole imaging dataset was exported as a *.bif6 file and subjected to multivariate analysis by means of principal component analysis (PCA) using plstoolbox (Eigenvector Research Inc., Wenatchee, WA) in Matlab (R2013a, MathWorks, Natick, MA). For PCA, the data were mean centered and scaled using Poisson scaling.

For statistical data analysis of spectral data, anatomical regions of the brain were assigned using the ROI feature implemented in the Surface Lab software (v 6.1, IONTOF) (Supplementary Fig. 2). The ROI’s were reconstructed with the predefined mass list using the Mass Explorer of the Surface Lab Software. Peak area values for all ROI and animals, respectively, were exported to MS Excel 2010. The statistical analysis was performed using SPSS v.23.0 (IBM Corp., Armonk, NY, USA). Comparisons between the groups were performed with Mann Whitney U test at a 95% confidence interval. Identification of mass peaks was done using tentative assignment by comparing the accurate mass to previously reported values in the literature^19^ as well as matching of isotopic distribution.. Statistical analysis for immunohistochemistry data was performed using GraphPad Prism^®^ (La Jolla, CA, USA). All data are expressed as mean ± standard error of the mean (SEM). Statistical differences presented here were calculated using an unpaired t-test. Brain lithium levels were analyzed using an ordinary one-way ANOVA followed by a Bonferroni post-hoc test. *p* < 0.05 was considered statistically significant.

## Results

### ToF-SIMS allowed identification of specific brain regions displaying differential distribution of lithium and other molecular species

**Figure 1:**
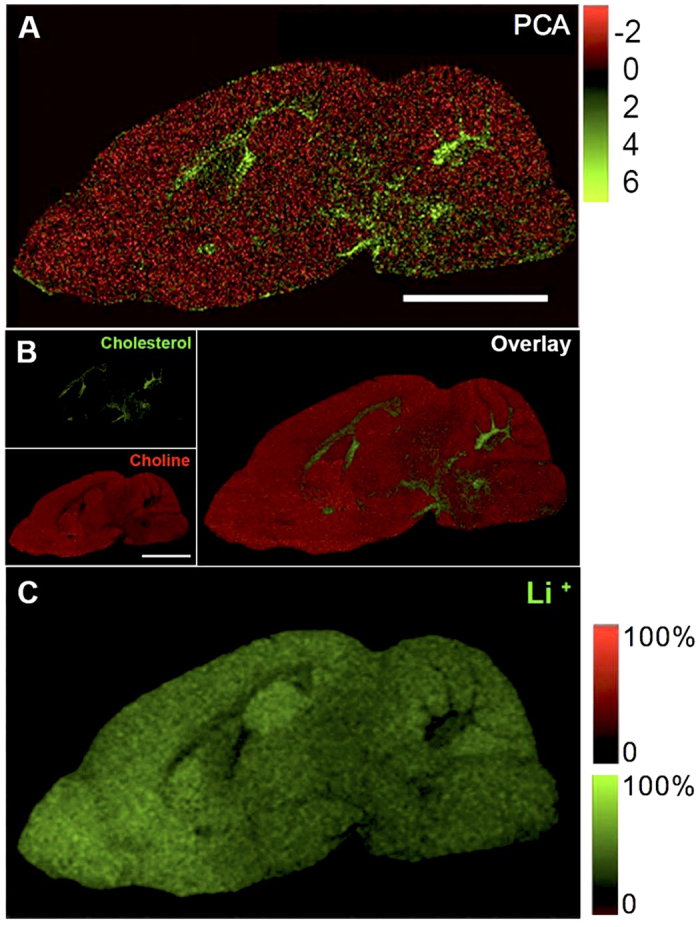
Multivariate Image analysis identified anatomical regions of interest. PCA and single ion images from one representative animal. (**A**) Score image for the first principal component obtained from PCA of the imaging data illustrating the major chemical differences of distinct anatomical regions. (**B**,**C**) From the corresponding loadings, the variables (m/z) that contribute the most to variance can be identified. These localize to different anatomical regions that can hence be delineated based on their chemical identity. These include, choline (**B** upper left), m/z = 104.1, [C_5_H_14_NO^+^], gray matter), cholesterol (**B** lower left), m/z = 369.33, [C_27_H_45_^+^], white matter) and the overlay (**B** on the right): choline (red) and cholesterol (green) and Li^+^ (**C**, m/z = 7). Scale bar = 2 cm.

To elucidate molecular changes in mice treated with lithium, different regions of interest (ROI) in the brain were identified and annotated based on the ToF-SIMS imaging data. For comprehensive analysis of the acquired ToF-SIMS data, a two-step strategy based on multivariate analysis of image data and ROI spectral data was applied. First, statistical analysis of image data by means of principle component analysis (PCA) was performed to outline anatomical regions of interest based on their biochemical identity^21^,^30^. This approach facilitated identification of major regions of the brain, where a clear distinction from adjacent anatomical regions could be achieved. While the score (i.e. eigenvalue) images (Fig. 1A) illustrate the variance over the analyzed area for a specific factor, the biochemical information behind these differences is contained in the loadings (eigenvectors). The variables with the highest positive or negative loading values and therefore the largest impact on the score values represent the most promising candidate compounds that show true localization to distinct regions of interest. In turn, these species can serve as biochemical identifiers for a certain anatomical region. Indeed, manual inspection of single ion images revealed that characteristic localizations were observed for chemical species that displayed the highest or lowest loading values. Here, PCA image analysis revealed a characteristic distribution of lipid fragments such as choline (m/z 104.1, [C_5_H_14_NO^+^]) found to be most prominent in the gray matter regions (Fig. 1B upper left) and cholesterol (m/z 369.33, [C_27_H_45_^+^]) that was in turn localized in the white matter regions (Fig. 1B lower left) as further highlighted by the overlay of the individual single ion distributions (Fig. 1B on the right). Furthermore a characteristic distribution of the lithium signal (m/z 7, [Li^+^]) appeared to be localized to gray matter regions (Fig. 1C).

### Lithium treatment revealed a neurogenic region-selective uptake in the juvenile mouse brain

**Figure 2:**
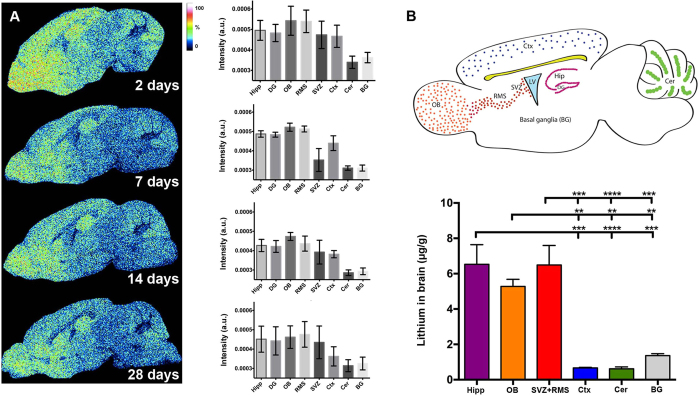
Region-selective lithium uptake following continuous treatment. (**A**) Single ion images from one representative animal per group for m/z 7 [Li^+^] at different time points. A characteristic decrease was observed in various anatomical regions at day 14 and 28 compared to day 2. This was particularly prominent in the cerebellum and cortex. (**B**) (Upper panel) Representative color-coded schematic image of a sagittal section of the mouse brain showing the ROIs identified by ToF-SIMS. Ctx = cortex, Cer = cerebellum, BG = basal ganglia, DG = dentate gyrus, Hip = hippocampus, LV = lateral ventricle, SVZ = subventricular zone, RMS = rostral migratory stream and OB = olfactory bulb. (Lower panel) ICP-AES quantification confirmed lithium-selective uptake in specific ROIs of the brain: SVZ+RMS, hippocampus and OB had significantly higher Li^+^ concentrations as compared to BG, cortex and cerebellum. ***p = 0.0001, ****p < 0.0001, *p*_OBvsBG_ = 0.0085, *p*_OBvscerebellum_ = 0.0015 and *p*_OBvscortex_ = 0.0017.

To rapidly reach a stable serum concentration, equivalent to therapeutical levels in the treatment of bipolar disorder, we administered a 4 mmol/kg loading dose of LiCl, followed by a 0.24% Li_2_CO_3_ chow for a period of 28 days. Based on the PCA score images, eight different anatomical regions of interest (ROI) were selected for analysis (Fig. 2A and B top panel, Supplemental Fig. 2). ToF-SIMS spectral data were extracted from the olfactory bulb (OB), the subventricular zone (SVZ), the rostral migratory stream (RMS), the hippocampus (Hip), the dentate gyrus (DG), the cerebral cortex (Ctx), the cerebellum (Cer) and basal ganglia (BG). Lithium had accumulated in all these brain regions already by day 2, and remained higher than in control brains at all time points (Mann-Whitney U test, p < 0.05) (Fig. 2A). Statistical analysis (Mann-Whitney U test, p < 0.05) revealed regional differences, as such that levels in the cerebellum and the basal ganglia were lower at all time points analyzed as compared to the olfactory bulb, cortex and the neurogenic zones (dentate gyrus, and rostral migratory stream). This observation was confirmed by ICP-AES quantification of the absolute amounts of lithium in each dissected region. The ICP data demonstrate that at 28 days, the highest lithium concentrations were found in the neurogenic regions, the subventricular zone and rostral migratory stream (6.49 μg/g), olfactory bulb (5.28 μg/g) and hippocampus (6.53 μg/g), as compared to non-neurogenic regions like the cerebellum (0.62 μg/g), cortex (0.66 μg/g) and basal ganglia (1.36 μg/g) (Fig. 2B).

### Serum lithium kinetic in the developing mouse

**Figure 3:**
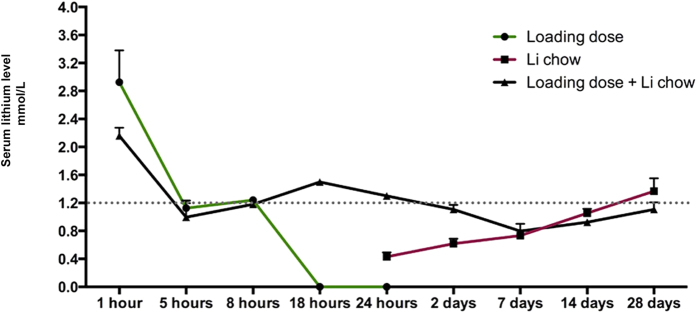
Serum lithium distribution at different time points. Measurements of the serum lithium levels at 1, 5, 8, 18 and 24 hours and 2, 7, 14, and 28 days after an initial loading dose of 4 mmol/kg LiCl and the onset of 0.24% Li_2_CO_3_ chow administration. After a single intraperitoneal injection the clearance of lithium serum levels is observed by 18 hours (green line), whereas with the chow the increase in serum lithium levels to therapeutically relevant concentrations did not occur until after 14 days (red line). In this study we combined a loading dose and the lithium chow (black line). In each time point n = 5.

Analysis of the serum lithium levels (Fig. 3) showed that the initial bolus injection caused a peak, followed by steady levels around 1.2 mmol/L at 5 and 8 hours after the onset of treatment, in the same range as the therapeutic levels in humans for bipolar disorder. After the initial loading dose alone, there were no detectable levels of lithium in the blood after 18 or 24 hours, in accordance with the clearance rates reported previously^31^. When oral administration alone was used (through the diet), it took 7–14 days before a steady level around 1.2 mmol/L was reached. However, when combining an initial intraperitoneal loading dose with continuous oral administration, a stable serum concentration level could be observed throughout the 28 days of treatment, making this a suitable strategy for this study from a pharmacokinetics point of view.

### Lithium treatment affected body weight gain and food intake

**Figure 4:**
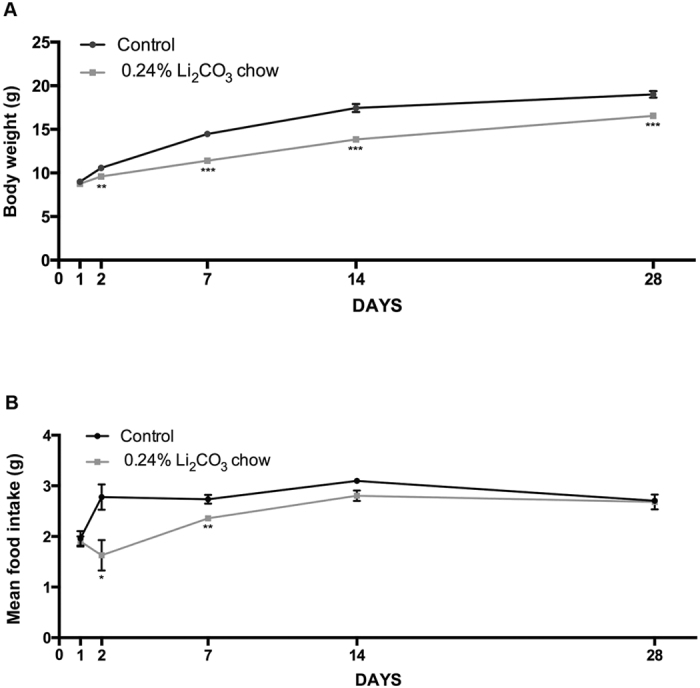
Chronic lithium treatment decreased body weight gain and food intake. (**A**) Graph showing the mean body weight in controls (black line) and in lithium-treated animals (gray line). Day 1 and 2 n = 18 control and n = 38 lithium, day 7 n = 18 control and n = 33 lithium, day 14 n = 12 control and n = 22 lithium, day 28 n = 12 control and n = 22 lithium. (**B**) Graph of the mean food intake per animal during lithium treatment. Significance was considered at p-values < 0.05. All data are presented as mean ± SEM.

Mice under lithium treatment displayed a lower body weight gain compared to the controls already after 2 days (**p = *0*.*0032*) from the onset of treatment and this lack of growth caused a persistently lower body weight at 7 days (***p < *0*.*0001*), 14 days (***p < *0*.*0001*) and 28 days (***p = *0*.*0001*) (Fig. 4A). Additionally, we observed that the food intake in the Li_2_CO_3_ chow group was lower than for the controls at 2 days (*p = *0*.*0446*) and within the first 7 days (**p = *0*.*0035*). However this difference in food intake gradually disappeared and reached normal levels at 14 and 28 days (Fig. 4B). Hence, although the food intake was restored to basal levels after 14 days, the body weight did not reach that of the control mice, even if a partial recovery was observed. In addition, we observed that lithium-treated animals suffered from polyuria and polydipsia, suggesting that this dose and mode of lithium administration predisposed these juvenile mice to nephrogenic diabetes insipidus.

### Lithium favored proliferation rather than neuronal differentiation in the neurogenic dentate gyrus of the hippocampus

**Figure 5:**
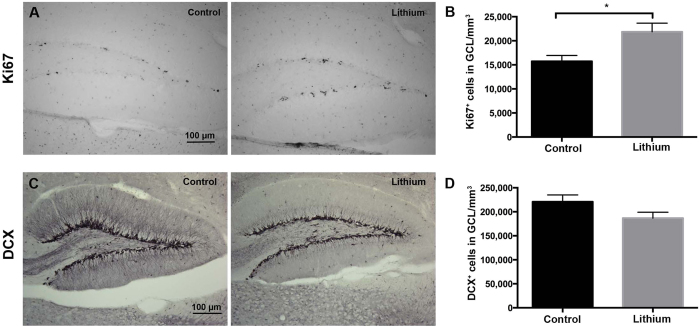
Lithium boosted hippocampal NSPC proliferation but not neuronal differentiation. (**A**) Ki67 immunoreactivity in the granule cell layer (GCL) of control and lithium-treated animals. In each group n = 6. (**B**) Bar graph showing the quantification of Ki67^+^ cells in the SGZ after 28 days of lithium treatment. Significance was considered at p-values < 0.05. (**C**) DCX immunoreactivity in the granule cell layer (GCL) of control and lithium-treated animals. (**D**) Bar graph showing the quantification of DCX^+^ cells in the GCL after 28 days from the onset of lithium treatment. In each group n = 6. Data are presented as mean ± SEM.

To glean insight into the effects of continuous lithium treatment on the generation and differentiation of newly born neurons, we quantified the density of proliferating (Ki67- positive) and doublecortin- (DCX-) positive cells in the DG (Fig. 5), the latter representing a transient population of both proliferating and post-mitotic immature cells which have committed to a neuronal lineage and display a migrating phenotype^32^. Here, we found a 1.34-fold increase in the density of proliferating cells after 28 days of lithium treatment compared to controls (*p = *0*.*0169*) (Fig. 5A and B). Further, we found that 28 days of lithium treatment did not alter the density of the immature neuronal cell type (*p* = *0*.*0997*) (Fig. 5C and D), suggesting that neuronal differentiation is not a major target or effect, at least not during continuous treatment with stable serum levels of lithium. To be able to exclude that this effect is lithium-specific and not age-dependent, we treated adult female mice with the same 0.24% Li_2_CO_3_ chow, identical to the dose previously reported^33^. We administered BrdU, a thymidine analogue that incorporates in dividing cells, the last five days of lithium treatment, enabling an estimate of the proliferative effect of lithium in the GCL of the adult hippocampus. Similar to the juvenile brain, we found a 1.66-fold increase in the density of proliferating cells after 14 days of lithium treatment compared to controls (***p = *0*.*0005*) (Supplementary Fig. 3A and B), but the density of the immature neuronal cell population showing immunoreactivity for DCX was not affected by lithium treatment when compared to controls (*p* = *0*.*6046*) (Supplementary Fig. 3C and D).

### Regional changes in lipid distribution associated with lithium uptake

In order to examine lithium-associated changes in brain lipids, changes of defined chemical species were investigated in the anatomical regions of interests previously delineated by image PCA of the ToF-SIMS data. The changes of peak intensity for each individual mass peak were compared between treatment groups and age-matched control groups within the respective ROI.

**Figure 6:**
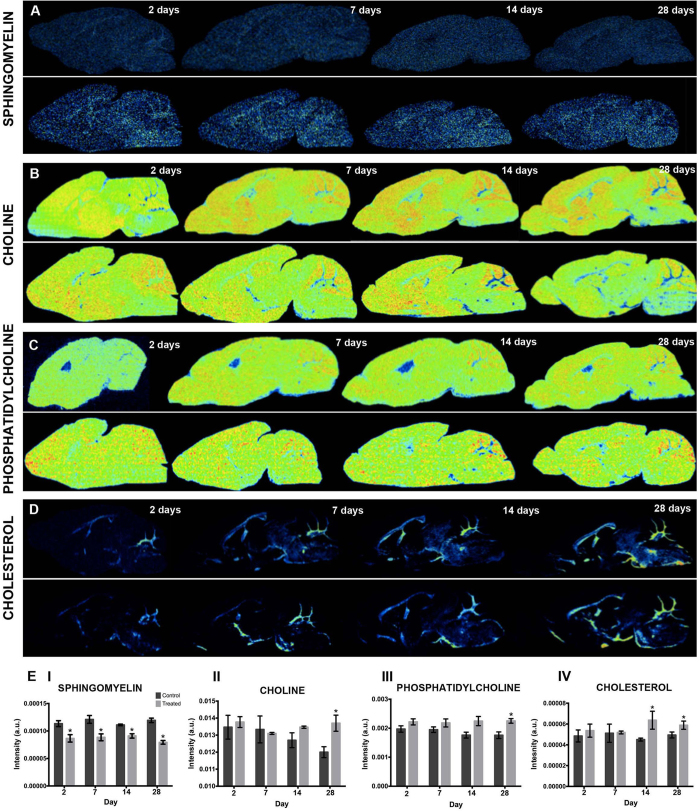
Lithium induced changes in region lipid levels. (**A**) Single ion images from one representative animal per group for sphingomyelin (m/z 264.26, SM, [C_17_H_30_NO^+^]), (**B**) choline (m/z 104.11 [C_5_H_14_NO^+^]), (**C**) phosphatidylcholine (m/z 224.11, PC, [C_8_H_19_PNO_4_^+^]) and (**D**) cholesterol (m/z 369.33, [C_27_H_45_^+^]) at different time points for treatment (top) and control (bottom) groups. (**E**) The levels of the individual lipid signals in cerebellar cortex of treatment groups were compared to the controls. (**E I**) Sphingomyelin SM (m/z 264.26) was significantly decreased for all the days in the lithium treatment groups. Conversely, choline (m/z 104.11) (**E II**) and phosphatidylcholine PC (m/z 224.11) (**E III**) showed significantly higher cortical levels at day 28. Similarly cholesterol (m/z 369.33) (**E IV**) displayed a significant increase on day 14 and 28.

**Figure 7:**
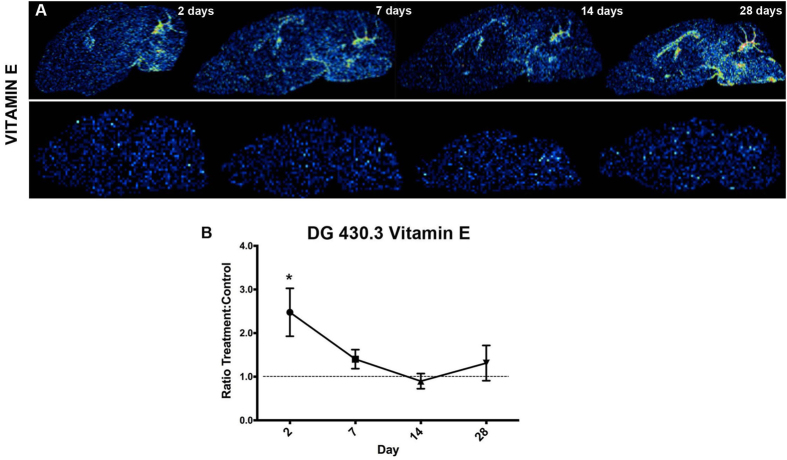
Vitamin E levels were transiently altered during lithium treatment. (**A**) Single ion images for vitamin E (m/z 430.33, alpha tocopherol, [C_29_H_50_O^+^]) at different time points during the lithium treatment (top) and in the control (bottom) group. (**B**) The levels of vitamin E (m/z 430.33) were elevated at day 2 in the dentate gyrus (DG), but leveled out at the later time points.

Four species, including m/z 262.10, m/z 264.26 (sphingomyelin headgroup, SM, [C_17_H_30_NO^+^]), m/z 281.08 and m/z 712.81) were found to be decreased in all the ROIs in the lithium-treated animals as compared to the age-matched controls. In detail, significantly lower values of brain-wide lipid levels were found for m/z 262.10 and m/z 264.26 (SM) at days 7–28 (Fig.6A, EI), and already on day 2–28 for m/z 281.08 and m/z 712.81. In contrast, peak intensity values of an unknown species with m/z 782.59 were found to be elevated in almost all the brain regions after day 7, except for the cerebellum. Statistical comparison between control and treatment groups for each of the time points revealed further regional changes for individual mass peaks that were distinct for different anatomical regions (Mann-Whitney, p < 0.05). Cortical levels of choline (m/z 104.11) and phosphatidylcholine (m/z 224.11, PC, [C_8_H_19_PNO^+^]) were found to be distinctly elevated on day 28 (Fig. 6B,C,EII and III). Similarly, the lipid fragment m/z 246.10 was elevated on day 28 in the cortex. Furthermore, cholesterol (m/z 369.33) was significantly elevated on day 14 in the cortex (Fig. 6D,EIV). Furthermore, analysis of the normalized levels of vitamin E (m/z 430.3) to the corresponding ROI average control signal and compared between the days (Mann- Whitney, p < 0.05) revealed an initial increase in hippocampal (hipp and DG) levels, however the effect leveled out over time (Fig. 7).

## Discussion

In this study we sought to characterize in a systematic fashion the effects of continuous lithium treatment in PND21 female mice for a period of 28 days, to shed light on the temporo-spatial lithium distribution as well as the effects on lipid distribution and neurogenesis in the juvenile mouse brain. For the first time, ToF-SIMS based high resolution imaging was employed for *in situ* measurement of brain wide lithium distribution. Previously, Li has been imaged *in vivo* using e.g. magnetic resonance imaging that allowed accurate quantification of brain wide Li levels however at sub cm resolution^34^. In contrast, ToF-SIMS is vastly superior in terms of spatial resolution that allows chemical imaging at submicron length scales, however at the cost of being limited for retrieving accurate quantitative information.

The here employed ToF-SIMS approach facilitated to delineate anatomical regions of interest based on their specific chemical profile (ROI). Here e.g. lithium and choline were found predominantly in the gray matter (Fig. 1B and C), whereas cholesterol localized in the white matter (Fig. 1B). PCA-based image analysis showed that lithium followed a spatial rather than a time- dependent pattern of distribution, in such that it varied in the subventricular zone but remained elevated in other neurogenic areas compared to basal ganglia, cerebellum and cortex (Fig. 2A). This selective uptake of lithium, as indicated by ToF-SIMS ion imaging, was further confirmed by absolute *ex vivo* quantification of lithium using ICP-AES on tissue extracts from dissected brain regions (Fig. 2B). As ToF-SIMS itself as a surface- sensitive technique cannot be considered to be quantitative^19^,^35^, the present *in situ* data and complemental *ex vivo* results highlight its suitability for relative quantification of spatial ion intensity distributions *in situ*^15^,^21^. Previously, it was shown that lithium accumulates in areas of the brain with higher cell density and our ToF-SIMS and ICP-AES analysis confirmed this^23^,^24^,^25^. A reason for lithium localizing to regions with higher cellular density could be that lithium uptake occurs chiefly at the cellular body level rather than at the axonal level as previous studies also hypothesized^24^,^25^. Overall, our findings contribute to a better understanding of lithium accumulation in the developing brain, providing not only a qualitative spatial lithium distribution as judged by ToF-SIMS but also supporting this with the more precise quantitative approach of ICP-AES. Based on our ICP-AES quantification of different ROIs, we estimate that brain lithium concentration would be in the range of 0.817–0.049 mmol/L, if we assume a tissue specific density of 1.04 g/ml as previously observed in a rodent study^36^.

Furthermore the paucity of reports^31^ on lithium serum levels and its pharmacokinetics in young and adults, prompt the generation of evidence for the correct lithium administration and maintenance paradigm that is pivotal for the comprehensive understanding of the yet elusive mechanism of lithium action in the brain. Here we show that in order to maintain a stable lithium level from the initiation of the treatment, the combination of a lithium-loading dose and lithium-enriched chow is preferable to the two singular approaches alone (Fig. 3).

As previously observed in rats and humans^37^,^38^,^39^, chronic but not acute lithium treatment often results in polyuria and polydipsia due to lithium decreasing the accumulation of cAMP and water reabsorption in the renal collecting tubules. In line with these findings, we reported here that young and adult female mice fed the lithium diet developed food aversion, leading to a slower bodyweight gain that partially normalized after 28 days, compared with control mice (Fig. 4A,B and Supplementary Fig. 3E and F). This slower weight gain was concurrent with excessive urination and increase water intake (polyuria and polydipsia), a well-known and transient side effect^37^,^40^.

Interestingly, this accumulation of lithium in neurogenic regions may be relevant for the positive effects observed on neurogenesis and other molecular processes relying on the generation and integration of new neurons in a pre-existing network^7^,^9^,^26^,^41^. Indeed, further investigation of the direct effects of lithium on neurogenesis revealed that a continuous treatment led to a significant, 1.34-fold increase in proliferation, as judged by quantification of Ki67^+^ cells in the GCL of the DG (Fig. 5B). However, no direct effect of lithium was observed on neural differentiation of newly born cells in the DG network, as judged by quantification of DCX^+^ cells (Fig. 5D). Our data are in line with a recent study where lithium, with the same dosing paradigm as used here, was found to target primarily the initial stage of progenitors, thereby enhancing the turnover of neural stem-/progenitor- cells, while it failed to translate into increased numbers of immature neurons^42^. More importantly, we found similar effects of lithium on body weight, food intake and neurogenesis in adult female mice (PND 49) exposed to continuous lithium treatment (Supplementary Fig. 3), suggesting that therapeutic levels (0.6–1.2 mmol) result in pro-proliferative but not pro-neurogenic effects as previously confirmed in our *in vitro* study^43^. This may be due to poor survival of the population of immature cells under lithium treatment. Nevertheless, it is well established that lithium *in vivo* decreases apoptosis through inhibition of glycogen synthesis kinase-3β activity, which translates into an upregulation of the anti-apoptotic molecules B-cell lymphoma protein-2, brain-derived neurotrophic factor, and β-catenin^44^,^45^. Due to the pro-proliferative and anti-apoptotic properties of lithium *in vivo*, it was unlikely that the immature neurons underwent apoptosis. In line with our recent *in vitro* findings^43^, we propose that lithium-induced neural progenitor cells, adopting the immature DCX phenotype, only transiently promoted the transition from G1 to S phase of the cell cycle. We further speculate that the rates of entry and exit across the DCX-expressing stage of the neuronal differentiation cascade may have been stimulated or accelerated before they developed into mature granule cells. We hypothesized that discontinuation of lithium treatment may be necessary to allow the increased number of proliferating cells to differentiate and integrate. These data confirmed that the effects of lithium *in vivo* on neurogenesis are independent of age.

As imaging mass spectrometry allows for comprehensive analysis of multiple chemical species *in situ*, we additionally analyzed changes in spatial intensity distribution levels of lipid species in lithium-treated animals compared with their age-matched controls. Here, we observed that several lipid species, including choline, sphingomyelin and cholesterol, displayed altered levels over time in different regions throughout the brain, suggesting that lithium targets lipid metabolism (Fig. 6). Sphingomyelin levels were decreased during the entire four-week period of lithium treatment (Fig. 6A and EI). On the other hand, the cortical levels of choline and phospatidylcholine (Fig. 6B,C,EII and EIII) as well as cholesterol (Fig. 6D and EIV) increased over time and were higher towards the end of the four weeks of the lithium treatment. Lipids are basic components of cellular membranes and are important in cell signaling, including neural stem cell differentiation^46^. Additionally, lipid metabolism is pivotal in ensuring life-long neurogenesis and targeting this metabolic pathway may hold great potential for therapeutic approaches to treat neurogenesis-related cognitive decline^47^. The free choline signal can originate from either phosphatidylcholine (PC) lipid species or acetylcholine. Increased choline can therefore be an indicator of neuronal cell integrity and proliferation^21^. Furthermore, both cholesterol and sphingomyelin support the formation of lipid rafts, which constitute functional units in membrane signaling and trafficking^48^. The mechanisms by which they operate are however still elusive, but there are indications that they may be involved in mediating cell signaling triggered by growth factors and cytokine receptors, ultimately supporting NSPC maintenance, polarization and differentiation^46^.

In addition, a more than 2-fold increase of vitamin E was observed in the dentate gyrus of lithium-treated brains at day 2 followed by stabilization at later time points (Fig. 7). Vitamin E (alpha-tocopherol) is a lipid-soluble alkylated phenol species with a strongly electrophilic group capable of efficiently scavenging free radical intermediates, making it a strong antioxidant and an efficient neutralizer of unstable lipid peroxy-radicals generated from polyunsaturated fatty acids^49^,^50^. It is known that vitamin E is highly expressed in the cerebellar region in the Purkinje cells^51^ and its antioxidant role in neurodegenerative diseases has been extensively investigated^52^. An initial increase in vitamin E in the DG as observed in this study may therefore indicate beneficial effects of lithium treatment in the developing brain.

Taken together, we demonstrate here that ToF-SIMS is a powerful approach to determine the spatial distribution of small molecules with sufficient resolution that allows for correlating chemical changes to discrete functional regions of the brain. This is the first study demonstrating differential distribution of lithium in the juvenile brain as well as its association with changes in lipid distribution and the known pro-neurogenic effects. It is noteworthy to mention that extending our approach to post-mortem analysis of tissue from patients treated with lithium may shed light on previously observed differential responsiveness to lithium in psychiatric conditions^53^. The reason for lithium preferentially distributing in confined brain regions is not known, however a previous study^54^ as well as our findings support the notion for lithium regulating the homeostasis of neurogenic areas, which contribute to a better understanding of the elusive mechanisms of lithium action in the brain. Finally we provide here evidence that lithium accumulation correlates with overt regional changes, thereby deepening the knowledge on how and why lithium may affect various brain functions.

## Additional Information

**How to cite this article**: Zanni, G. *et al*. Lithium Accumulates in Neurogenic Brain Regions as Revealed by High Resolution Ion Imaging. *Sci. Rep.* **7**, 40726; doi: 10.1038/srep40726 (2017).

**Publisher's note:** Springer Nature remains neutral with regard to jurisdictional claims in published maps and institutional affiliations.

## Supplementary Material

Supplementary Information
