# Supplementary material for: Lithium Accumulates in Neurogenic Brain Regions as Revealed by High Resolution Ion Imaging
Source: Sci Rep. 2017 Jan 18;7:40726. doi: 10.1038/srep40726 (PMC5241875; doi:10.1038/srep40726)

Supplemental Information

Lithium Accumulates in Neurogenic Brain Regions as Revealed by High Resolution Ion Imaging

Giulia Zanni [1,2], Wojciech Michno [3], Elena Di Martino [1], Anna Tjärnlund-Wolf [2], Jean Pettersson [4], Charlotte Elizabeth Mason[1], Gustaf Hellspong [1], Klas Blomgren [1,5] * & Jörg Hanrieder [3, 6,7] *

[1] Karolinska Institute, Department of Women's and Children's Health, Karolinska University Hospital, Stockholm, Sweden

[2] Center for Brain Repair and Rehabilitation, Institute of Neuroscience and Physiology, Sahlgrenska Academy at the University of Gothenburg, Gothenburg, Sweden

[3] Department of Psychiatry and Neurochemistry, Sahlgrenska Academy at the University of Gothenburg, Mölndal, Sweden

[4] Department of Chemistry-Biomedical Centre, Uppsala University, Uppsala, Sweden.

[5] Department of Pediatric Oncology, Karolinska University Hospital, Stockholm, Sweden.

[6] Department of Chemistry and Chemical Engineering, Chalmers University of Technology, Gothenburg, Sweden

[7] Department of Molecular Neuroscience, UCL Institute of Neurology, University College London, UK

Supplementary figure 1. Dry-to-wet weight ratio of different brain region of interest (ROI). Quantification of the weight of different brain areas (in mg): SVZ +RMS (9.65 ± 3.151), Hipp (8.25 ± 1.984), OB (7.25 ± 1.261), Cer (47.2 ± 9.6), BG (28.8 ± 1.766) and Ctx (77.7 ± 4.121). Data are presented as mean ± SD

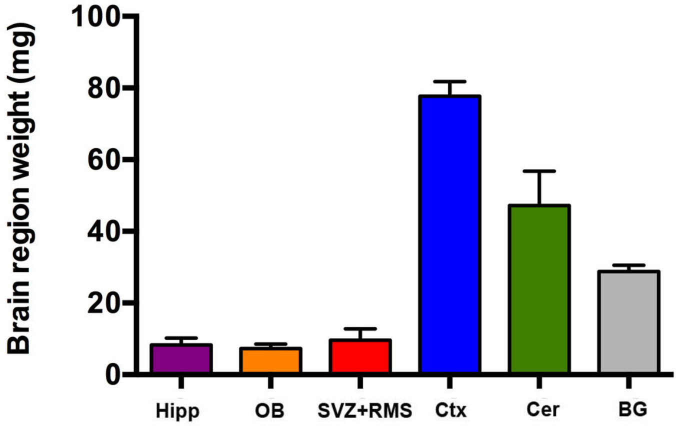

Supplementary figure 2. Annotation of different brain region of interest (ROI) for SIMS data analysis. The regions were annotated following outlining of major anatomical features by distinct ion signals (choline, m/z 86.7: grey matter and cholesterol, m/z 369.3: white matter). The following ROIs were outlined: Ctx = cortex, Cer = cerebellum, BG = basal ganglia, DG = dentate gyrus, Hipp = hippocampus, SVZ = subventricular zone, RMS = rostral migratory stream and OB = olfactory bulb. Scalebar: 2cm

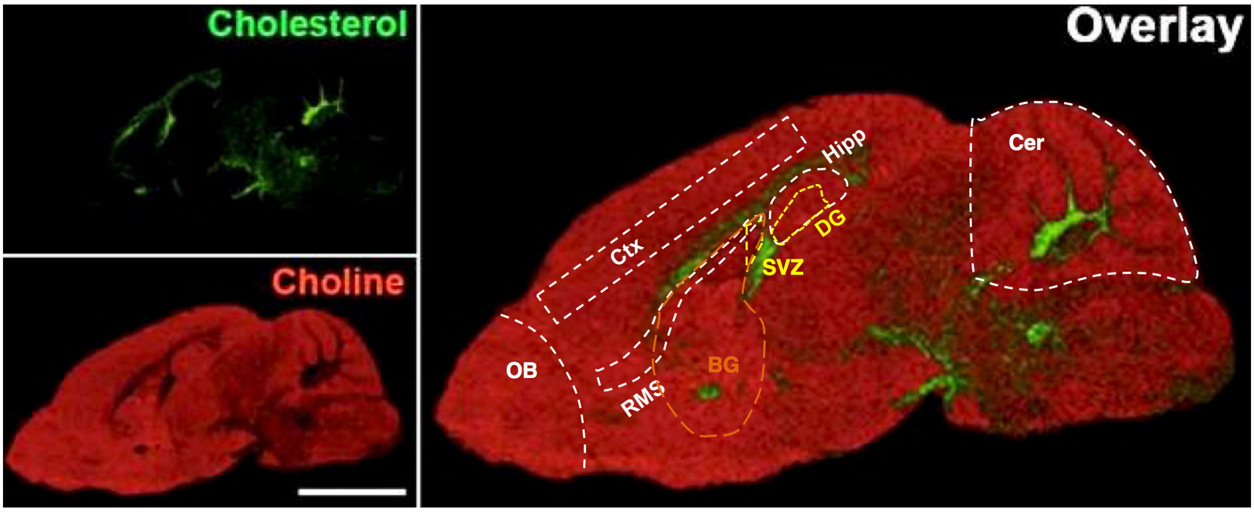

Supplementary figure 3. Adult female mice display comparable effects to young female mice in lithium response. (A) BrdU immunoreactivity in the granule cell layer (GCL) of control and lithium-treated animals. In each group n=5. **(B)** Bar graph showing the quantification of $BrdU^+$ cells in the SGZ after 14 days of lithium treatment. Lithium increased proliferation of 1.66-fold compared to control adult female mice, ****p= 0.0005*. **(C)** DCX immunoreactivity in the granule cell layer (GCL) of control and lithium-treated animals. **(D)** Bar graph showing the quantification of DCX^+ cells in the GCL after 14 days from the onset of lithium treatment. In each group n=5. Neuronal differentiation, as judged by DCX immunoreactivity, did not result in detectable effects, *p= 0.6046*. **(E)** Graph showing the mean body weight in controls (black line) and in lithium-treated animals (gray line). The effects of lithium on body weight are evident at 14 days **p=0.0155*. **(F)** Measurements of the serum lithium levels showing that lithium levels in the serum of adult female mice reached therapeutically relevant concentrations of 0.7 mmol/L after 28 days of treatment. Data are presented as mean ± SEM.

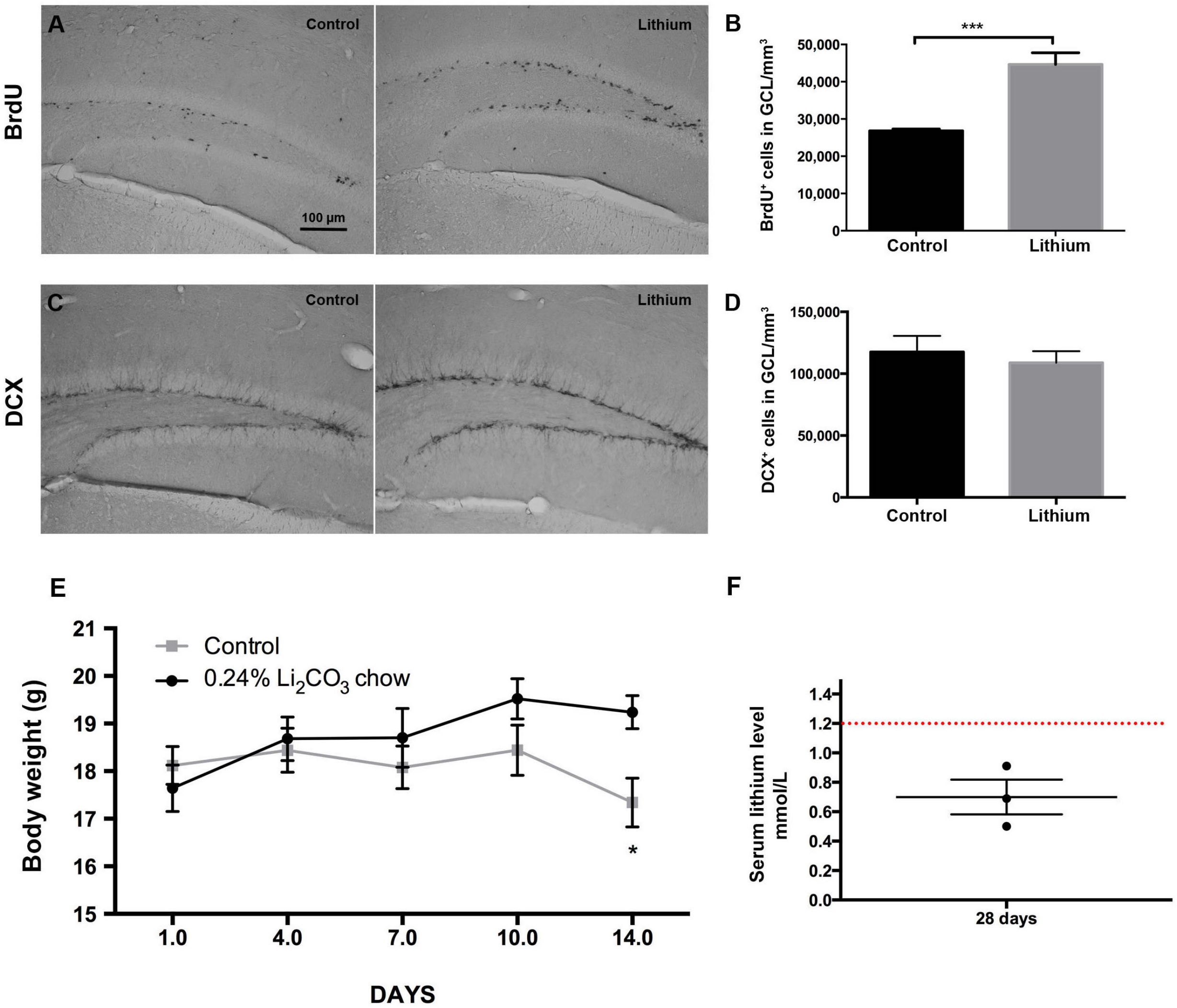

Supplement: Supplementary Information [file srep40726-s1.pdf]
